# Evolutionary shift detection with ensemble variable selection

**DOI:** 10.1186/s12862-024-02201-w

**Published:** 2024-01-20

**Authors:** Wensha Zhang, Toby Kenney, Lam Si Tung Ho

**Affiliations:** Department of Mathematics and Statistics, Dalhousie University, Nova Scotia, Canada

**Keywords:** Evolutionary shift detection, Ornstein-Uhlenbeck model, LASSO, Trait evolution, Ensemble method, Phylogenetic comparative methods, ELPASO

## Abstract

Abrupt environmental changes can lead to evolutionary shifts in trait evolution. Identifying these shifts is an important step in understanding the evolutionary history of phenotypes. The detection performances of different methods are influenced by many factors, including different numbers of shifts, shift sizes, where a shift occurs on a tree, and the types of phylogenetic structure. Furthermore, the model assumptions are oversimplified, so are likely to be violated in real data, which could cause the methods to fail. We perform simulations to assess the effect of these factors on the performance of shift detection methods. To make the comparisons more complete, we also propose an ensemble variable selection method (R package ELPASO) and compare it with existing methods (R packages $\ell$1ou and PhylogeneticEM). The performances of methods are highly dependent on the selection criterion. $\ell$1ou+pBIC is usually the most conservative method and it performs well when signal sizes are large. $\ell$1ou+BIC is the least conservative method and it performs well when signal sizes are small. The ensemble method provides more balanced choices between those two methods. Moreover, the performances of all methods are heavily impacted by measurement error, tree reconstruction error and shifts in variance.

**Supplementary Information:**

The online version contains supplementary material available at 10.1186/s12862-024-02201-w.

## Introduction

Understanding the evolutionary process of species is an important task in phylogenetic comparative studies. Felsenstein [1] is the first to introduce Brownian Motion (BM) to model the evolution of a continuous trait. BM models have been used in many evolutionary studies, such as flower size evolution [2], genome size evolution [3], the spread of HIV-1 in central Africa [4], and mammalian life history traits [5]. Hansen [6] proposed to use an Ornstein-Uhlenbeck (OU) processes to model evolution with natural selection. Unlike the BM process, the variance of the OU process in traits is bounded, which is more realistic [7]. Therefore, OU processes are now widely used in many evolutionary studies, including character displacement in Lesser Antillean Anolis Lizards [7], and HIV-1 heritability [8].

Butler and King [7] formulate the multiple optima OU model for adaptive evolution in which optima differ between branches, and remain constant along an evolutionary path until discrete events where changes in selective regime occur. They use hypothesis testing to test whether the optima are different between groups. The changes can be modeled as shifts in the parameters of the OU processes. The shifts in optima are believed to be correlated with abrupt environmental changes [9, 10]. For example, Jaffe et al. [11] investigate the relationship between the difference in optimal body sizes and habitat changes for turtles. Therefore, by detecting shifts in optima based on observed traits, we can get knowledge of unobserved historical environmental changes and better understand the evolutionary process of species. Using statistical models to detect the evolutionary shifts, where the shifts have occurred and the size of the shifts, becomes an important problem to be solved.

There are some existing approaches to address this problem. Uyeda and Harmon [12] propose a Bayesian framework to detect shifts in the selective optimum of OU models. However,the computation cost of Bayesian approaches is relatively high. We will focus on frequentist approaches in this paper. Ho and Ané [13] illustrate the limitation of traditional model selection criteria (AIC, BIC) in the shift detection task and propose to use forward-backward selection with modified BIC [14]. Khabbazian et al. [15] formulate the shift detection problem into a variable selection problem and combine the OU model with LASSO to detect the shift points, which they implement in the $\ell$1ou R package. Bastide et al. [16] develop a maximum likelihood estimation procedure based on the EM algorithm (implemented in the R package PhylogeneticEM).

Recently, ensemble methods have been widely applied to variable selection problems [17–19]. Ensemble feature selection can add more diversity to selected variables and produce robust variable selection results [20]. In this paper, we propose an ensemble variable selection method for shift detection and compare it with existing methods implemented in the PhylogeneticEM and $\ell$1ou packages. We have implemented our method in a new R package, ELPASO (Ensemble LASSO for Phylogenetic Analysis of Shifts with OU). It is available at https://github.com/WenshaZ/ELPASO.

The main target of this paper is to compare the detection performances of different methods under the influence of various factors. Many factors influence the detection performances of different methods, including different numbers of shifts, different shift sizes, where a shift occurs on a tree (near the root; in the middle; near the tip), and the types of phylogenetic structure. Furthermore, the violations of the model assumptions could bring challenges to the shift detection task. In order to describe the influence of the factors, we perform simulations under various scenarios. The simulation results show that the most conservative methods perform well when the signal sizes are large for selecting a limited number of false positive shifts. In contrast, the least conservative methods perform well when the signal sizes are small for selecting a larger number of true positive shifts. How conservative a method is mostly depends on the selection criterion. pBIC [15] is a more conservative criterion than BIC. $\ell$1ou+pBIC is usually the most conservative method and performs well in cases with large signal sizes. $\ell$1ou+BIC is the least conservative method and performs well when signal sizes are small. The ensemble methods provide more balanced choices between those two methods. From the simulations with model mis-specification, we show that the measurement error, tree construction error and shift in diffusion variance will bring challenges to the shift detection task, leading to the failure of the existing methods.

The remainder of this paper is organized as follows. “Shift detection for trait evolution models” section introduces the problem formulation of the shift detection task as a variable selection problem, and presents the methodology of $\ell$1ou, PhylogeneticEM and our ensemble method. “Simulations without model mis-specification” section shows our simulation results for the different methods on simulated data under assumed OU models. In order to better evaluate the performance, we use three different criteria: true positive and false positive shifts detected, predictive log-likelihood on a test data set, and Adjusted Rand Index. We discuss the effect of number of shifts, shift size, shift position and tree shape on the performances of detection methods. “Simulations with model mis-specification” section compares the methods when the model assumptions are not satisfied. Previous simulation studies often ignore this scenario. However, in practice, the model assumptions are usually violated. Finally, “Conclusion” section provides conclusions and discussion.

## Shift detection for trait evolution models

### Trait evolution models

The trait evolution process on a phylogenetic tree describes the changes in traits through generations. Each species has a certain value of the trait. And the trait values of different species are correlated because of their shared ancestry which is represented by a phylogenetic tree. We only observe the trait values at the tips of the tree. In this paper, the tree is assumed to be separately estimated from sequence data, and is treated as known.

Trait evolution models are used to model how the trait values change over time. Brownian Motion and Ornstein-Uhlenbeck are two commonly used models to model the evolution of continuous traits. Let $\textrm{Y}$ denote the vector of observed trait values at the tips, $Y_i$ as the trait value of taxon *i*. These two models assume that conditioning on the trait value of a parent, the evolutionary processes of sister species are independent. So we only need to specify the model on one branch. For a single branch, we let *Y*(*t*) denote the trait value at time *t*.

#### Brownian motion model

Felsenstein [1] proposed to use Brownian motion (BM) to model the evolution of continuous traits over time. Trait values of sister lineages start at the trait value of their most recent common ancestor and evolve independently following a BM model. The result of this model is that the correlation between the trait values of two species depends only on the evolution time they shared. Under this model, the observed trait values $\textrm{Y}$ follow a multivariate Gaussian distribution. For an ultrametric tree of height 1, each $y_i$ has mean $\mu _0$ and variance $\sigma ^2$, and the covariance between $y_i$ and $y_j$ is $\sigma ^2 t_{ij}$, where $t_{ij}$ is the shared evolution time between species *i* and *j*.

#### Ornstein-Uhlenbeck model

The variance of the BM model is unbounded, which is considered unrealistic. The OU model [6], on the other hand, incorporates a selection force that pulls the trait value toward a selective optimum $\theta$. This model is preferable to the BM model because of its more realistic assumptions. An OU process *Y*(*t*) is defined by the following stochastic differential equation

$$\begin{aligned} dY(t)=\alpha [\theta (t)-Y(t)]dt+\sigma dB(t) \end{aligned}$$

where *dY*(*t*) is the infinitesimal change in trait value; *B*(*t*) is a standard BM; $\sigma ^2$ measures the intensity of random fluctuation; $\theta (t)$ is the optimal value of the trait at time *t*; and $\alpha \ge 0$ is the selection strength. When $\alpha = 0$, the OU process is the same as a BM. We assume that $\alpha$ and $\sigma$ are constant.

### Shift detection as a linear model selection problem

For the OU model, the assumption that the optimal value $\theta (t)$ is constant throughout the tree is not realistic, as different trait values are suited for different environments and evolutionary strategies. A more practical model allows the optimal value $\theta (t)$ to shift at certain positions on the tree. Hansen [6] proposed a heterogenous OU model to allow different optimal values on different branches. Shifts are noncontinuous changes in the optimal value during the evolution process. A shift on a branch of the phylogenetic tree would influence all the species under that branch. Our goal here is to find the positions of shifts and estimate the changes in optimal trait value, $\theta$, at the shifts. We assume that any shift in optimal value only occurs at the beginning of the branch, therefore the optimal value is constant along a single branch. Let $\theta _b$ denote the optimal value on branch *b* and $t_{\textrm{start}(b)}$ the age of the beginning of branch *b*. Let *T* denote the age of the root node. We only consider ultrametric trees in this shift detection task. Let $\textrm{pa}(b)$ denote the parent edge of *b* and $\textrm{end}(b)$ the end node of *b*. Thus, $\triangle \theta _b = \theta _{\textrm{pa}(b)}-\theta _b \ne 0$ means that a shift in optimal value occurred on branch *b*.

Let $\textbf{Y}$ denote the observed trait values at the tips, $Y_i$ denote the trait value of taxon *i* and $Y_0$ denote the trait value of the root node. Under the OU process, $\textbf{Y}$ follows a multivariate normal distribution. Conditional on the initial trait value, the mean of each random variable $Y_i$ is [6]:

$$\begin{aligned} E(Y_i) = Y_0e^{-\alpha T}+\sum \limits _{b \in \textrm{path}(\textrm{root},i)}\left( e^{-\alpha t_{\textrm{end}(b)}}-e^{-\alpha t_{\textrm{start}(b)}}\right) \theta _b \end{aligned}$$

and the covariance between $Y_i$ and $Y_j$ is $\sigma ^2 e^{-\alpha d_{ij}}(1-e^{-2\alpha t_{ij}})/(2\alpha )$.

Therefore, if we let $Y_0$ follow the stationary distribution for the initial process (normal with mean $\theta _0$ and variance $\sigma ^2/(2\alpha )$) then $E(Y_i)=\theta _b$ and the covariance is $\Sigma _{ij}^{(\alpha )}=\sigma ^2 e^{-\alpha d_{ij}}/(2\alpha )$ [21] where $t_{ij}$ is the shared time between species *i* and *j*, and $d_{ij}$ is the distance between taxa *i* and *j*. To transfer the shift detection problem into a regression problem, we can rewrite the mean of $Y_i$ as [15]:

1 $$\begin{aligned} E(Y_i){} & {} = Y_0e^{-\alpha T}+\sum \limits _{b \in \textrm{path}(\textrm{root},i)}\left( e^{-\alpha t_{\textrm{end}(b)}}-e^{-\alpha t_{\textrm{start}(b)}}\right) \theta _0 \nonumber \\{} & {} \qquad +\sum \limits _{b \in \textrm{path}(\textrm{root},i)} \sum \limits _{b' \in \textrm{path}(\textrm{root},b)}\left( e^{-\alpha t_{\textrm{end}(b)}}-e^{-\alpha t_{\textrm{start}(b)}}\right) \triangle \theta _{b'} \nonumber \\{} & {} =Y_0e^{-\alpha T} + (1-e^{-\alpha T})\theta _0 \nonumber \\{} & {} \qquad + \sum \limits _{b' \in \textrm{path}(\textrm{root},i)} \sum \limits _{b \in \textrm{path}(b',i)}\left( e^{-\alpha t_{\textrm{end}(b)}}-e^{-\alpha t_{\textrm{start}(b)}}\right) \triangle \theta _{b'} \nonumber \\{} & {} = Y_0e^{-\alpha T} + (1-e^{-\alpha T})\theta _0 + \sum \limits _{b' \in \textrm{path}(\textrm{root},i)}(1-e^{-\alpha t_{\textrm{start}\ {b'}}})\triangle \theta _{b'} \end{aligned}$$

Let $\beta _0 = Y_0e^{-\alpha T} + (1-e^{-\alpha T})\theta _0$ and $\beta _b = (1-e^{-\alpha t_{b}})\triangle \theta _{b}$. In this way, the shift detection problem under the OU model can be converted to a linear model selection problem. The trait values at tips can be written as:

$$\begin{aligned} \textbf{Y} =\beta _0\textbf{1}+\sum \limits _b\beta _b \mathbf {X_b}+{\epsilon } \end{aligned}$$

where $X_b$ is a vector defined by $X_{bi}=0$ if taxon *i* is not under branch *b*, and $X_{bi}=1$ if taxon *i* is under branch *b*, and $\epsilon$ follows a normal distribution with mean 0 and covariance matrix $\Sigma ^{(\alpha )}$. The main task is to select the branches that have $\beta _b \ne 0$.

### l1ou

Khabbazian et al. [15] propose a phylogenetic LASSO method to detect shifts in optimal trait value under OU models. To remove the influence of the covariance matrix, they conduct a transformation before model selection:

$$\begin{aligned} \Sigma _{\alpha }^{-1/2}\textbf{Y} =\beta _0\Sigma _{\alpha }^{-1/2}\textbf{1}+\Sigma _{\alpha }^{-1/2}\textbf{X}{\beta }+\Sigma _{\alpha }^{-1/2}{\epsilon } \end{aligned}$$

Where ${\beta }$ denotes the vector of $\beta _b$ and $\textbf{X}$ denotes the design matrix, the $b\text {th}$ column of $\textbf{X}$ is $\mathbf {X_b}$. After data transformation, the error terms $\Sigma _{\alpha }^{-1/2}\epsilon$ become a vector of independent standard normal random variables. The LASSO solution is to minimize the least squares with $\ell 1$ penalty. The loss function is given by

$$\begin{aligned} \frac{1}{2}\left\| \Sigma _{\alpha }^{-1/2}\textbf{Y}-\beta _0\Sigma _{\alpha }^{-1/2}\textbf{1}-\Sigma _{\alpha }^{-1/2}\textbf{X}{\beta }\right\| ^2+\lambda \Vert \beta \Vert _1 \end{aligned}$$

They use the package lars to estimate $\beta$ for every $\lambda$ value, and conduct backward selection based on the model selection criterion pBIC (see “Model selection criteria” section for more details about pBIC) using the models selected for each $\lambda$ value as a starting point. They then use the same criterion to select from among the models found for different values of $\lambda$.

The above process is based on a given $\alpha$ value. Khabbazian et al. [15] use the following procedure to obtain the estimation of $\alpha$. Firstly, they set $\alpha =0$ and run the variable selection for this value of $\alpha$. They then refit $\alpha$ with the selected variables. They repeat the selection step for the new $\alpha$, and choose the model with the best criterion score from among these models. Because of the use of LASSO, the computation speed is faster than previous shift detection tools, including SURFACE [22] and bayou [12]. Their implementation of the method is available in the R package $\ell$1ou.

### PhylogeneticEM

Bastide et al. [23] introduce a framework which treats phylogenetic analysis as a missing data problem, allowing the usage of the EM algorithm. They set ${\tau } = (\sigma ,\alpha , {\beta })$ as the vector of all the parameters to estimate, and ${\textbf {X = (Z,Y)}}$ as the trait values of both internal and external nodes. ${\textbf {Z}}$ is the vector of trait values of internal nodes, ${\textbf {Y}}$ is the vector of trait values of external nodes.

They assume the number of shifts is fixed and use the EM algorithm to estimate the parameters by maximizing the log likelihood $\textrm{log}{p_\tau (\textbf{Y})}$. The EM algorithm is based on the decomposition:

$$\begin{aligned} \textrm{log}{p_\tau (\textbf{Y})} = E_Z[\textrm{log}{p_\tau (\textbf{Z,Y})}|\tau ]-E_Z[\textrm{log}{p_\tau (\mathbf {Z|Y})}|\tau ] \end{aligned}$$

The difficulty with the maximization, in this case, comes from the fact that the locations of shifts on the branches are discrete variables. They used a Generalized EM (GEM, Dempster et al. [24]) to conduct the maximization. The complexity for this is $O(n^k)$ where *k* is the number of shifts.

The above process is based on the assumption that the number of shifts *k* is fixed. They estimate the parameters with $k = 1, ...., K$, where *K* is the given maximum number of shifts. They then conduct model selection on *k* based on penalized least squares:

$$\begin{aligned} \text {PLS} = \left( 1+\frac{\text {pen}(k)}{n-k-1}\right) \sum \limits _{i=1}^p\Vert Y_i-\hat{Y_i}\Vert ^2 \end{aligned}$$

where $\hat{Y}_i$ is the predicted trait value of taxon *i* given by the model with *k* shifts. The penalty term $\textrm{pen}(k)$ if given by

$$\begin{aligned} \text {pen}(k) = A\frac{n-k-1}{n-k-2}\text {EDkhi}\left[ k,n-k-2,\frac{(k+1)^2}{|S_k^{PI}|}\right] \end{aligned}$$

where $|S_k^{PI}|$ denotes the number of parsimonious identifiable sets of locations of k shifts and *A* is a constant which the authors fixed at 1.1 based on simulation results. The EDkhi function [25] is defined as follows: Let

$$\begin{aligned} \textrm{DKhi}(D,N,x)= \frac{1}{\text {E}(X_D)}\text {E}\left[ \left( X_D-x\frac{X_N}{N}\right) _+\right] \end{aligned}$$

where *D* and *N* are positive integers, $X_D$ and $X_N$ are independent chi-squared random variables with *D* and *N* degrees of freedom respectively. Then, for $0<q\le 1$, $\textrm{EDkhi}(D,N,q)$ is defined as the unique solution to

$$\begin{aligned} \text {Dkhi}[D,N,\text {EDkhi}[D,N,x]] = q \end{aligned}$$

Baraud et al. [25] show that the penalty used here bounds the risk of the selected variables, and gives non-asymptotic guarantees. The implementation of this method is available in the R package PhylogeneticEM.

### Ensemble method

The framework of the ensemble variable selection model for shift detection consists of two phases. Firstly, we apply LASSO on a number of random subsamples of the transformed data. For each subsample, we obtain a ranking of the variables based on the largest penalty $\lambda$ for which the variable is selected by LASSO. We aggregate the rankings from each subsample into an overall variable ranking. Secondly, we use this ranking as a basis for a variable selection method.

**Fig. 1:**
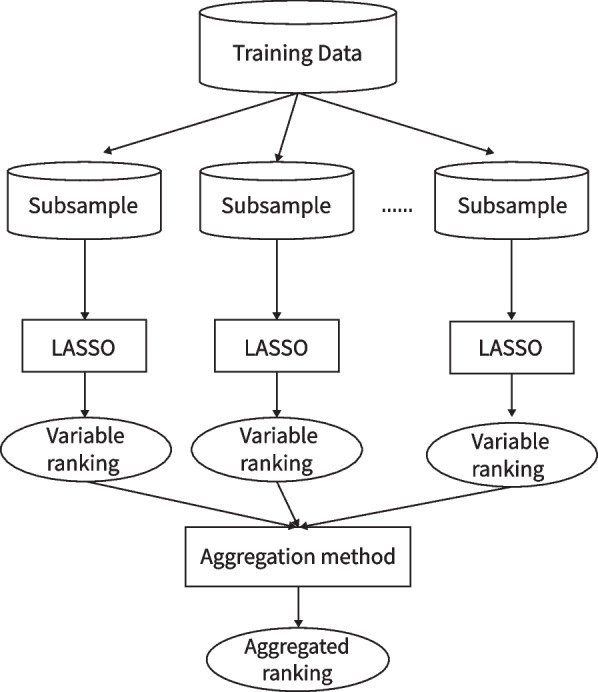
The model structure of ensemble method for shift detection

The foundation of ensemble learning is to combine the results of multiple models. The idea is that combining the results of several models will obtain better results by reducing the model variance and bias. Bagging and boosting are the two most commonly used ensemble models. There has been substantial recent work on the use of ensemble models for feature selection. Bolón-Canedo and Alonso-Betanzos [26] summarize the different types of ensemble methods that are used in feature selection. We here use a homogenous scheme for the ensemble. That is, we firstly take subsamples from the training dataset, then apply LASSO to each subsample. LASSO provides a solution path by varying the penalty size. Therefore, for each subsample, a variable ranking is produced where variables are ranked in decreasing order of the largest penalty for which they are selected. Aggregating the ranking sequences from all the subsamples, we can get the overall ranking for all the variables. The process is shown in Fig. 1.

There are several choices for how we aggregate the rankings from the different subsamples into a single overall ranking. For example, geometric mean, arithmetic mean, median, and so on. It is possible to apply these aggregation methods either to the ranking or to the values of the penalty $\lambda$. We suggest using the first quartile of the ranking to aggregate the results because the first quartile is robust to outliers. In a few subsamples, a shift may be ranked very low, possibly because the taxa that distinguish it from other shift positions are not included in the subsample. In these cases, the rank might be very large, and therefore have a large influence on the geometric or arithmetic mean. Furthermore, using the first quartile can improve our ability to distinguish between shifts and surrogates. It is common for only one of the surrogate variables to be ranked highly, and the shift to have a low rank. This can cause the median rank of the true shifts to be low. However, if the number of surrogates is not excessive, the first quartile of the rank will usually be high.

After obtaining the aggregated ranking, we use stepwise selection to choose the final combination of variables. Forward selection, backward selection, and forward-backward selection are potential approaches. Forward selection starts with the null model, and sequentially adds variables in the ranked order, starting with the highest ranked, as long as the model score is better than the previous model. Backward selection starts with the full model, and sequentially removes variables in rank order, starting with the lowest ranked, as long as the model score after removing each variable is better than the previous model. forward-backward selection consists of one forward selection pass followed by one backward selection pass, starting with the model selected by forward selection. Firstly include the variables that improve the model score then remove the variables whose removal further improves the model score. From the simulation results, forward-backward selection performs best.

Our procedure to estimate $\alpha$ and $\sigma ^2$ is similar to $\ell$1ou.

1. Fit a null BM phylogenetic regression model on the dataset. Get the initial estimate of $\sigma ^2$.
2. Use $\alpha =0$ and the $\sigma ^2$ value from the first step to calculate the covariance matrix and apply the ensemble method variable selection procedure.
3. Fit the phylogenetic regression model with the selected variables in Step 2 and get new estimates of $\alpha$ and $\sigma ^2$.
4. Repeat Step 2 and Step 3 once more.
5. Select the model with the best model criterion score.

### Model selection criteria

For all the models, a criterion is used to conduct model selection for the number of shifts. AIC and BIC are most the commonly used criteria in model selection problems. Ho and Ané [13] showed that using AIC as the criterion may lead to model overfitting. Khabbazian et al. [15] present a new criterion pBIC including a phylogenetic correction. The traditional BIC is given by:

$$\begin{aligned} \textrm{BIC}(M_k) = -2\textrm{loglik}(M_k)+(2k+3)\textrm{log}(n) \end{aligned}$$

where *n* is the number of taxa, *k* is the number of shifts selected, $M_k$ is the estimated model. $2k+3$ is the number of parameters: each shift location and magnitude is counted as a parameter and there are 3 general parameters ($\beta _0$, $\alpha$, and $\sigma$). The phylogenetic BIC proposed by Khabbazian et al. [15] is given by:

2 $$\begin{aligned} \textrm{pBIC}(M_k){} & {} = -2\textrm{loglik}(M_k)+2k\textrm{log}(2n-3)+2\textrm{log}(n) \nonumber \\{} & {} \qquad +\textrm{log}\ \text {det}\left( \left( X_{M_k}^{\hat{\alpha }}\right) ^{T}v \Sigma _{\alpha }^{-1}X_{M_k}^{\hat{\alpha }}\right) \end{aligned}$$

where $X_{M_k}^{\hat{\alpha }}$ is the matrix $X^{\alpha }$ with only the columns corresponding to the k selected shifts, and *v* is the observed trait variance. The penalty for the shift position is $2k\textrm{log}(2n-3)$. The penalty for shift magnitudes and the intercept are shown in the last term. PhylogeneticEM uses penalized least squares for model selection; details are in “PhylogeneticEM” section.

## Simulations without model mis-specification

We conduct simulations to compare PhylogeneticEM, $\ell$1ou (pBIC/BIC) and ensemble LASSO (pBIC/BIC). The most direct method for comparison is to compare how many true shifts the methods detect and how many wrong shifts are selected. However, the OU model is not completely identifiable [13, 15, 16], and even if the selected shifts are not equivalent to the true model, a good argument can be made that selecting a close surrogate shift is preferable to failing to select the shift at all. In these cases, the true positive versus false positive curve might misrepresent the performance, since neither method has a true positive, but the method that selects the surrogate has a false positive, and so is deemed to have performed worse, even though selecting the close surrogate is arguably more correct. Therefore, we include two more measurements in comparison: predictive log-likelihood and Adjusted Rand Index [27]. The idea of predictive log-likelihood is to compare the prediction accuracy on test data, of the selected models from different methods. Adjusted Rand Index evaluates how similar the clustering of the selected model is to the clustering of the true model. We can get a more comprehensive understanding of the characteristics, strengths, limitations of the methods by combining the three different measurements.

**Fig. 2:**
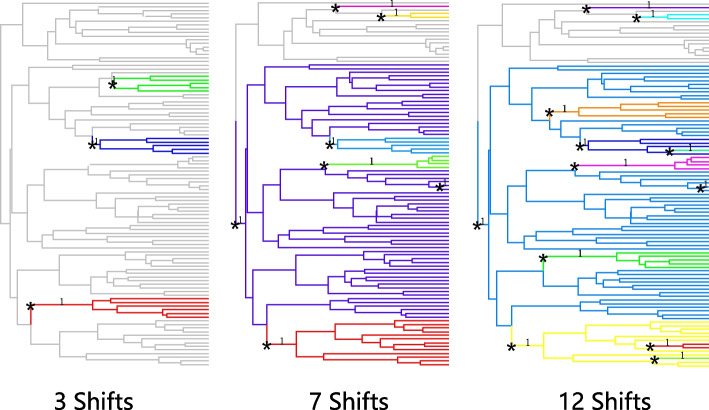
Tree used in simulations to compare the precision and recall of different methods. The shifts positions are indicated by asterisks. Different colours indicate different optimal values for the trait

We simulate a number of scenarios with varying numbers of shifts and signal sizes. We simulate datasets under OU models along the 100-taxon Anolis lizards’ tree [10]. We compare the methods on scenarios with 3, 7, or 12 shifts. Data were simulated according to the shifts in Fig. 2. For each scenario, we set $\alpha = 1$ and $\sigma ^2 = 2$ so that $\frac{\sigma ^2}{2\alpha } = 1$. We simulate under eight true signal sizes: $\beta =0.2, 1, 1.5, 2, 2.5, 3,5,7$ and 10. In each simulation, all shifts have the same value of $\beta$. For simplicity, only the results of simulations with 7 shifts are presented in the plots in the main text. The results of simulations with 3 and 12 shifts have similar conclusions and are presented in Supplemental Material. We also show the results for only some $\beta$ values in the true positive versus false positive plot and ARI plot. The results for omitted $\beta$ values are similar to the results shown.

### True positive versus false positive

We first compare the methods by true positive versus false positive curves. True positive is the number of true shifts detected by the model. False positive is the number of shifts that are not simulated but which are wrongly detected by the model. If two models have similar false positive values, the one with a higher true positive value is considered to have a better performance. If one model has a higher true positive and higher false positive than another, there is no obvious conclusion about which model is better. It becomes a trade-off problem between precision and recall.

**Fig. 3:**
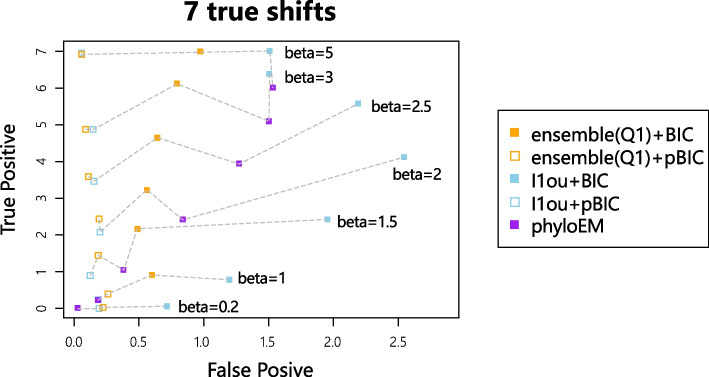
True positive numbers versus False positive numbers with 7 shifts

Figure 3 shows the average true positive versus false positive curve from 200 simulations in each scenario. Each point in Fig. 3 represents the mean of true positive and mean of false positive values. From the simulation results, $\ell$1ou+pBIC is usually the most conservative method, with both lowest true positive and false positive. For example, in the simulation of 7 shifts and $\beta =2$, $\ell$1ou+pBIC on average detects under 2 shifts. $\ell$1ou+BIC is usually the least conservative method. Ensemble LASSO provides more balanced choices between those two methods. In most simulations, ensemble LASSO with BIC and pBIC have both higher true positive and false positive compared to $\ell$1ou+pBIC and have both lower true positive and false positive compared to $\ell$1ou+BIC. Furthermore, in some situations, ensemble LASSO methods have a better performance compared to $\ell$1ou. For example, in the simulation of 7 shifts and $\beta =2$, ensemble LASSO+pBIC has higher true positive and lower false positive compared to $\ell$1ou+pBIC and ensemble LASSO+BIC has higher true positive and lower false positive compared to $\ell$1ou+BIC. PhylogeneticEM is even more conservative than $\ell$1ou+pBIC when the signal sizes are small. It performs well where there are only 3 true shifts. However when the number of shifts and the coefficient sizes are large enough, PhylogeneticEM performs poorly compared to other methods, including more false positive and fewer true positive variables.

### Predictive log-likelihood

**Fig. 4:**
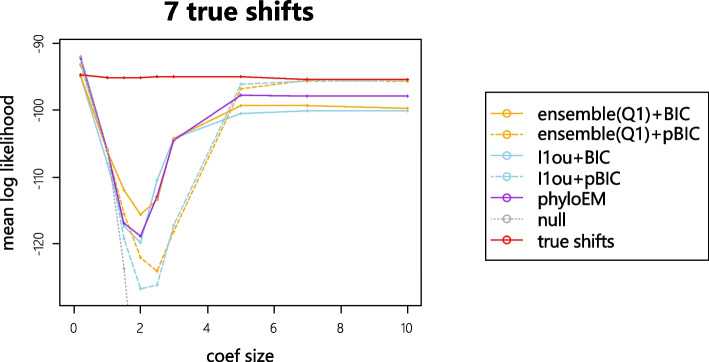
The mean log likelihood on 1000 test datasets

For each simulation, we generate 1000 test datasets and 200 training datasets. The test datasets are generated using the same tree with the same shift positions and values. We calculate the mean of log likelihood values over 1000 test datasets using the estimated shifts from training sets. For all the methods, we only use the selected shift positions and re-estimated the shift magnitudes on the training set. When the results of a method give a higher predictive log-likelihood value, it indicates that the method performs better at predicting the trait values. Figure 4 shows the mean of average log likelihood values over 1000 test datasets with different numbers of true shifts and coefficient sizes.

From the simulation results, when the size of coefficients are very small or very large, methods with pBIC have a higher prediction log likelihood value. When coefficient sizes are very small, methods with pBIC are very strict and tend to select nearly no shifts. In these scenarios, the signal sizes are so small that the null model has a higher prediction likelihood compared to the true model. When coefficient sizes are very large, all the methods can detect almost all the true shifts, while the methods with BIC might include more false positive shifts. Conversely, when the coefficient sizes are in the middle of the range, methods with BIC have a better performance in terms of prediction accuracy. PhylogeneticEM is quite conservative, with high predictive log-likelihood when the signal sizes are small. In most cases, the performance of PhylogeneticEM is between that of the pBIC and BIC methods. The difference between the ensemble method and $\ell$1ou is smaller than the difference between BIC and pBIC, and which method performs better varies between scenarios.

### Adjusted rand index

An alternative approach to assess the accuracy of the chosen shifts is to compare the induced grouping of species based on the shifts. We used Adjusted Rand Index (ARI, [27]) between the clustering of tips of the true model and the clustering of the estimated shifts to evaluate the model performances. The Rand index proportional to the number of pairs in agreement between two clusterings. The ARI is a scaled and centred version of the Rand index so that identical clusterings give an ARI of 1 and the expected ARI of two random clusterings is 0.

**Fig. 5:**
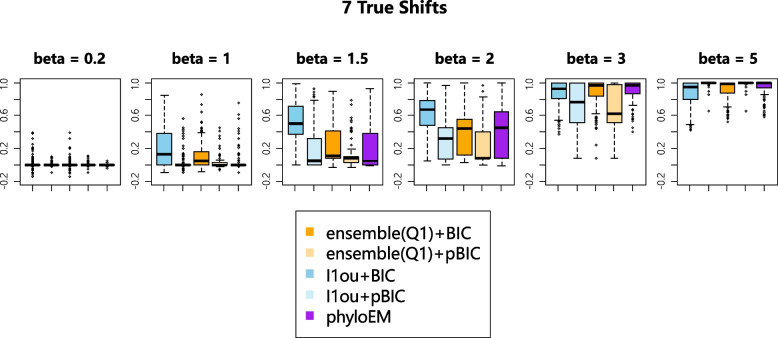
ARI with 7 true shifts

Figure 5 shows the ARI comparison of the different methods with 3, 7, and 12 shifts. ARI shows a similar result to the prediction log likelihood. When the signal sizes are small, the methods with BIC have a better performance. When the signal sizes are large enough, the methods with pBIC have a higher ARI score. PhylogeneticEM has low ARI score when the signal sizes are small and good performances when the signal sizes are in the middle. Based on true positive versus false positive numbers, PhylogeneticEM has poor performance with 7 and 12 shifts, but its predictive log-likelihood and ARI are comparable to other methods. This means that the shifts estimated by PhylogeneticEM might not be the exact true shifts, but they give similar trait predictions and trait clustering results.

### Shift position

**Fig. 6:**
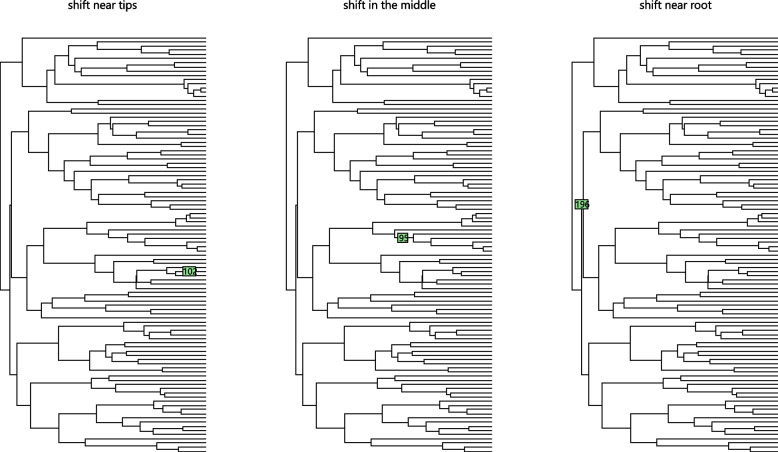
Shifts in different positions of tree

In this section, we study the influence of shift position. Intuitively, different shift positions influence different numbers of taxa and have different evolution time for the taxa so the results might be different. Indeed the pBIC criterion is designed specifically to account for the effect of shift position. We perform simulations with only 1 shift in different positions on the tree (Fig. 6). We perform simulations with $\beta =1,5,10$, $\alpha =1$ and $\sigma ^2=2$.

**Fig. 7:**
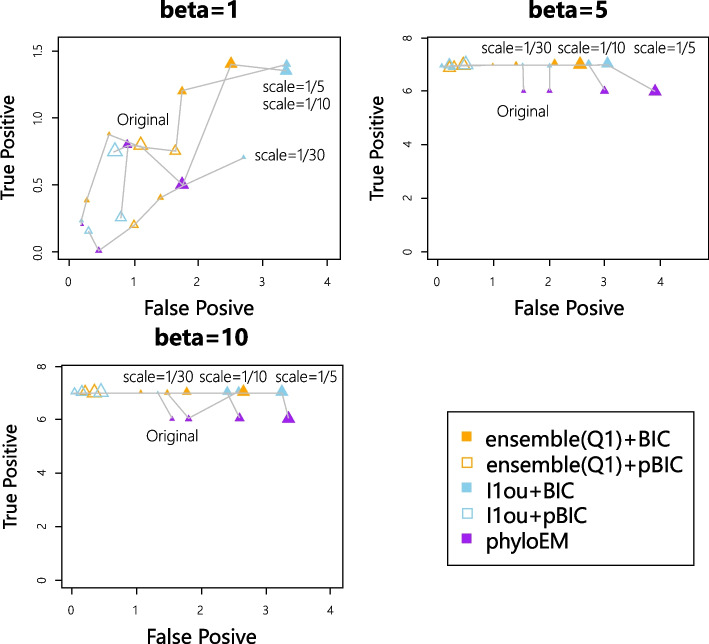
True positive versus false positive with different shift positions

Figure 7 shows the true positive versus false positive curves of different shift positions. When the coefficient size is very large, all the methods can detect the true positive shift correctly, regardless of its position. When the coefficient size is not large enough, the shift near the root is the easiest one to detect. All the methods have higher true positive values compared to shifts in other positions. The result is in line with common sense — shifts near the root influence a large group of taxa and the evolution time after the shift is longer, so the shift might have a larger influence on the trait values, making it easier to detect. However, shifts near leaves are easier to detect compared to shifts in the middle based on the simulations for $\beta =5$. And for $\beta =5$, ensemble LASSO+BIC performs better than $\ell$1ou+BIC at detecting the shift near the root or the shift near the leaves. PhylogeneticEM has a good performance for detecting the shift near the root but has worse performance for detecting the shift near the leaves and the shift in the middle.

### Different types of tree

**Fig. 8:**
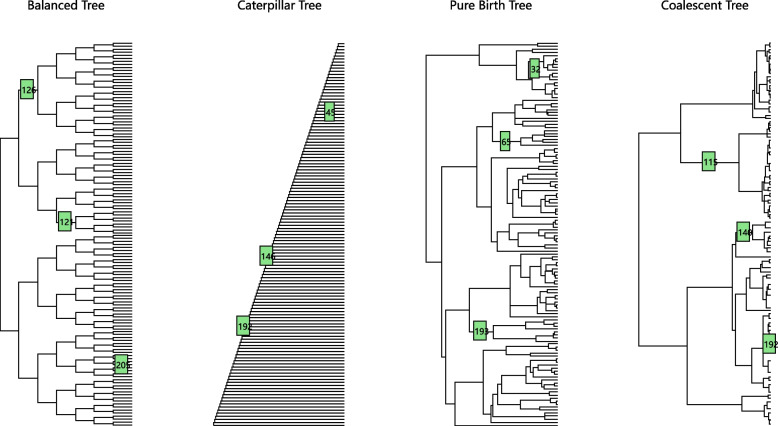
Four different types of tree

In this section, we compare the method performances on shift detection tasks on different types of phylogenetic trees. We mainly consider 4 types of tree: balanced tree, caterpillar tree, pure birth tree and coalescent tree. We generate these 4 types of trees with 128 taxa and 254 edges. Figure 8 shows the generated tree and simulated shifts for each type of tree. In this simulation, there are 3 shifts on each tree.

**Fig. 9:**
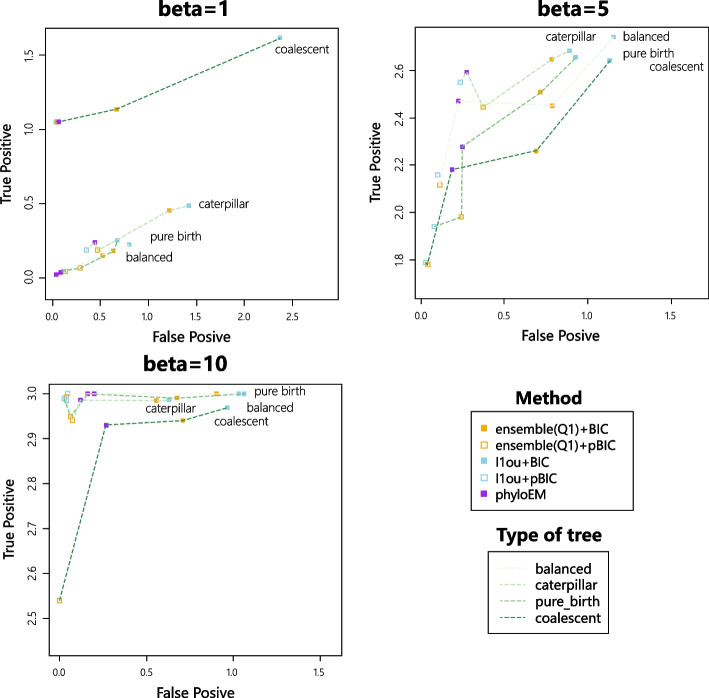
True positive versus false positive with different types of trees

Figure 9 shows the true positive versus false positive curves for different types of trees. Interestingly, the shifts in the coalescent tree are the easiest to detect when the coefficient size is small and the most difficult to detect when the coefficient size is large. However, the results might also be influenced by the shift setting of the experiment. For the result of coalescent tree, the shift 115 is easy to detect and the shift 192 is difficult to detect. For other types of tree, generally speaking when the coefficient size is in the middle of the range, the shifts on the caterpillar tree are the easiest to detect, and then the balanced tree, then the pure birth tree and finally the coalescent tree.

From the simulation results, the performances of methods are highly dependent on the selection criterion. The phylogenetic Bayesian information criterion (pBIC) is more conservative than the Bayesian information criterion (BIC). When the signal sizes are small, the methods using pBIC struggle to detect any signal, leading to poor detection results. And when the signal sizes are large, nearly all the methods are able to detect the true positive shifts while the methods using BIC include too many false positive signals. Therefore, when the signal sizes are small, the methods using BIC have better performance and when the signal sizes are large, the methods using pBIC have better performance. $\ell$1ou+pBIC is usually the most conservative method and $\ell$1ou+BIC is the least conservative method. Thanks to the ensemble of numerous selection results, ensemble LASSO provides more balanced choices between those two methods. Moreover, different positions of shifts and different types of trees result in selection tasks with different difficulties. The shifts near the root are the easiest to detect. The shifts on the caterpillar tree are the easiest to detect, and then the balanced tree, then the pure birth tree and finally the coalescent tree.

## Simulations with model mis-specification

In this section, we study the performance of the methods in situations where the model assumptions from “Shift detection for trait evolution models” section are not met, or where the parameter $\alpha$ is misestimated. We study three common misspecified situations: measurement errors in the trait values; a misspecified tree and the diffusion variance $\sigma ^2$ not constant throughout the tree. Parameter misestimation is not usually considered in model misspecification studies, but because $\ell$1ou and ensemble LASSO use a very crude method for estimating the rate of mean reversion, it is possible that this estimate will be far from the truth, so it is important to see how they are influenced when this happens. We therefore also simulate a situation in which we deliberately mis-estimate $\alpha$. The three misspecified scenarios are all common difficulties. Some traits are difficult to measure accurately, resulting in additional variance in the measured values. The tree is estimated from sequence data, usually via a model which is much simpler than the true evolutionary process, and therefore, the estimated phylogenetic tree is subject to both bias and sampling variance. Finally, an environmental shift in evolutionary history might lead not only to a shift in the optimal value, but also to a shift in variance. If shifts in variance occur, it might also bring difficulties for the shift detection methods.

From the simulation results, $\ell$1ou and ensemble LASSO are robust to the misestimation of $\alpha$. Therefore, although both methods only uses 2-3 iterations to estimate the $\alpha$ value, it doesn’t affect the detection of shifts even if the estimation is not perfect. However, the performances of all the shift detection methods are heavily impacted by measurement error, tree reconstruction error and shifts in variance. Because of the noise brought by these factors, the methods have difficulty detecting the true positive shifts and often include many false positive shifts. Moreover the incorrect tree might prevent convergence of the estimation of $\alpha$ in $\ell$1ou and ensemble LASSO. Further research about improving the shift detection performance under these challenging scenarios would be an interesting topic for future studies.

### Measurement errors

It is common for the measurement of traits to be subject to errors. These errors may impact the shift detection methods, which assume that the trait values are measured perfectly. In this section, we simulate additive Gaussian measurement error $N(0,\sigma _e^2)$ for the trait value of each species. When $\sigma _e^2$ is larger, the size of the measurement error is larger.

**Fig. 10:**
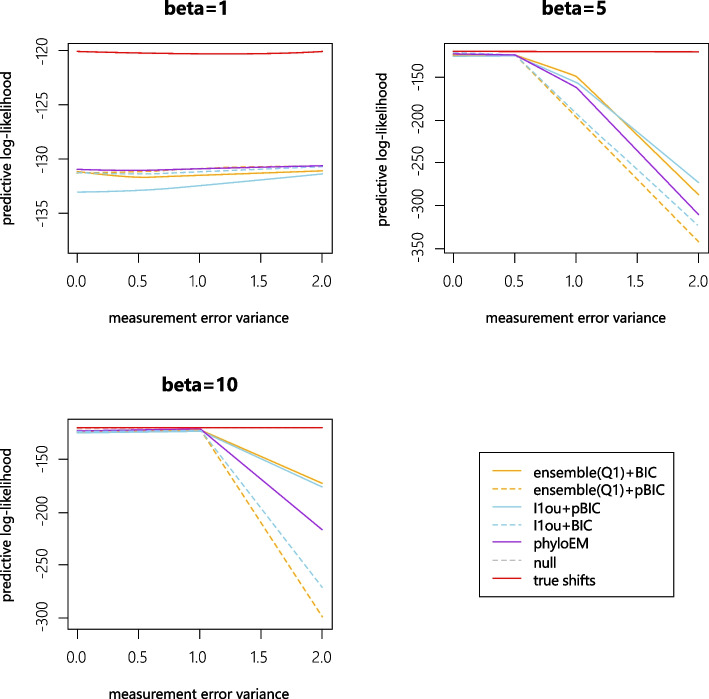
Average test log likelihood with parameters estimated from training data without measurement error using the shifts selected from training data with measurement error

Because of the measurement errors in the training data, the true shift model sometimes has a lower log-likelihood than the null model. If we use the training data with measurement error to estimate the parameters of selected shifts, the parameters will be far from the real values and thus introduce errors while calculating the predictive log-likelihood. Since our purpose is to identify the true shifts, predictive log-likelihood using training data with measurement error to estimate the parameters is not an ideal way to assess the selected shifts. Therefore, we also compare the prediction log-likelihood of the shift detection methods using the training data with measurement error to select shifts, but with parameters estimated from training data without measurement error. Figure 10 shows the results of log likelihood with parameters estimated from training data without measurement error. Prediction log-likelihood results with shift magnitudes estimated from data with measurement error are shown in Supplemental Material. The plot shows that the measurement error will influence the accuracy of shift detection. The loss of accuracy increases with the number of shifts. The performance of all the methods worsens with measurement error. Methods based on BIC are more robust to measurement error when the signal strength is strong, and perform less well when the signal strength is weak. The performance of PhyloEM is generally between the performance of methods using BIC and methods using pBIC. The difference between the performance of $\ell$1ou and the ensemble method is much smaller than between methods using BIC, and methods using pBIC. When the signal is not so strong ($\beta =1$), even a relatively small measurement error severely impacts performance.

### Incorrect tree

**Fig. 11:**
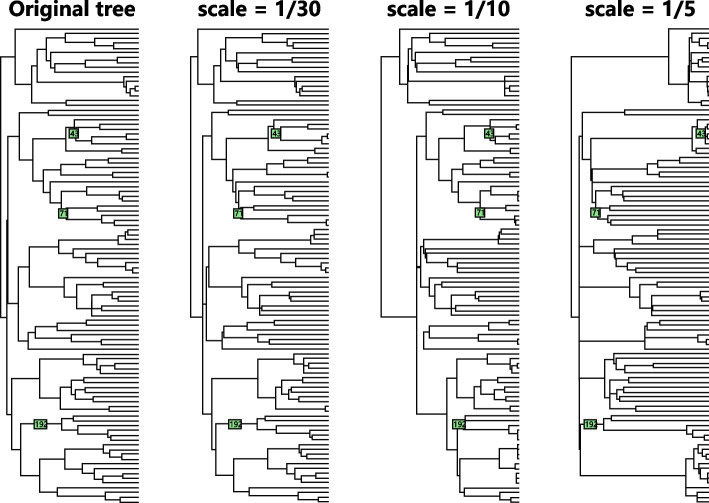
Regenerated tree with different beta

Another possible violation of the model assumptions is that we use a wrong tree to do the shift detection. There are two possible errors in the analyzed tree: wrong topology or wrong branch lengths. In the case of an incorrect topology, it is not always clear that there is a meaningful way to define “true shifts” in a false tree. Therefore, we will focus on the case with incorrectly specified branch lengths. We simulate data from a tree with different branch lengths and apply the methods using the original tree. We use a gamma distribution to randomly generate each internal branch length. The mean of each branch length is the original branch length. When the scale parameter is larger, the generated tree has larger difference from the original tree. In order to generate ultrametric trees and keep the total tree depth the same as the original tree, the external branches are generated by the original tree depth minus the tree depth of the starting node of each external branch. The internal branches are generated with a depth first order. If the depth of one internal branch is larger than the given tree depth, this branch length is resampled. We generate 3 trees with $scale = 1/30,1/10,1/5$ as shown in Fig. 11.

**Fig. 12:**
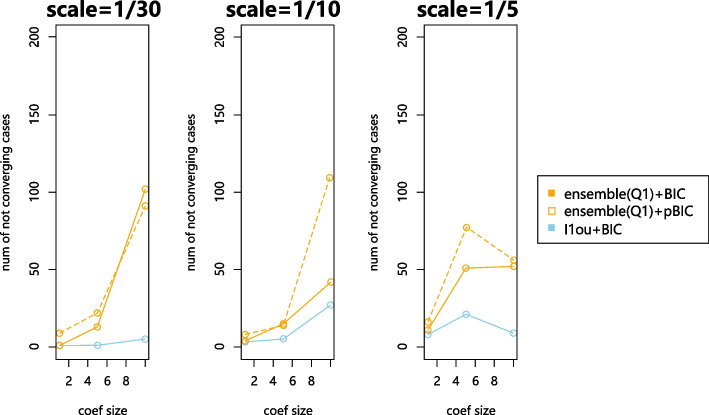
Number of non-converging cases when analyzing on a misspecified tree

Firstly, we find that this violation of assumptions can cause convergence problems while iterating the methods to estimate $\alpha$ in $\ell$1ou or the ensemble method. This is a problem we observe for real data in the Anolis Data. Figure 12 shows the number of cases where the estimated alpha does not converge in 10 iterations out of 200 simulations. The convergence problems seem to particularly affect the ensemble method but are also present for $\ell$1ou. For the non-converging simulations, we present results for the best $\alpha$ value attempted (in terms of our model selection criterion).

**Fig. 13:**
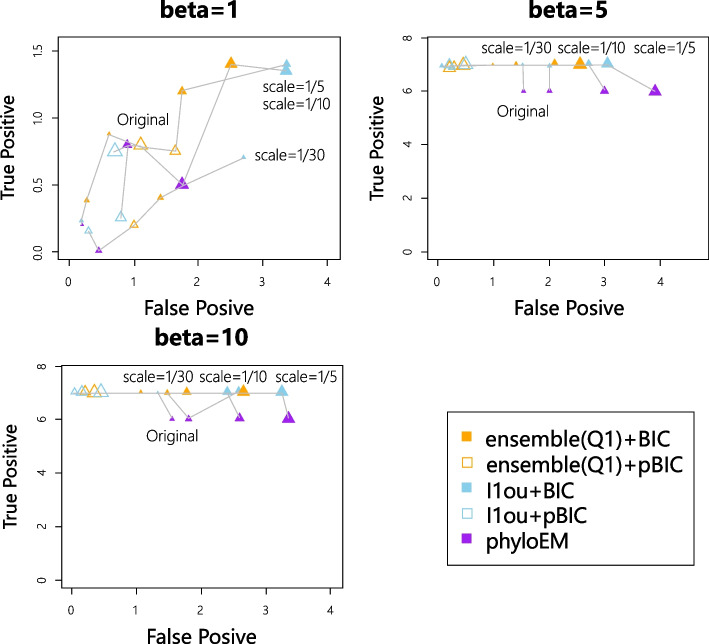
True positive versus false positive with applying methods on misspecified trees (larger triangles show larger difference with the original tree)

**Fig. 14:**
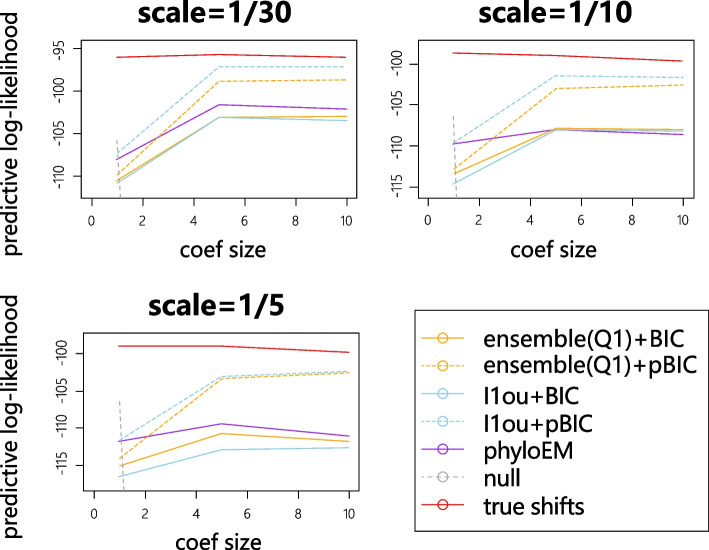
Average test log likelihood with applying methods on misspecified trees

Figure 13 shows the average number of true and false positives for each misspecified tree. Figure 14 shows the prediction log-likelihood. We see that generally speaking, the difference between the real tree and the analyzed tree will worsen the performance of the methods by either lowering the true positive rate or increasing the false positive rate. However, the methods are relatively robust to this misspecification. PhyloEM appears to be most influenced by the misspecification, becoming less conservative when the tree is misspecified. This increases both the true positive rate and the false positive rate. The predictive log-likelihood on test data is fairly robust to the tree misspecification. In some exceptional cases, the methods perform better when the tree is misspecified. For example, for simulations with 7 or 12 true shifts, nearly all the methods had higher true positive rates when the tree was most misspecified (scale parameter = 1/5) than for the original tree.

### Misestimation of $\alpha$

Recall from “Shift detection for trait evolution models” section that both $\ell$1ou and ensemble use a very rough method to estimate $\alpha$. This could lead to the estimated $\alpha$ values being very bad. In this section, we investigate the extent to which misestimation of $\alpha$ can impact the variable selection results. In this simulation, we use $\alpha = 1$ to generate data and use different fixed values of $\hat{\alpha }$ values $\left( 10^{-4},10^{-3},...,10^2\right)$ in the methods and compare the model performances.

**Fig. 15:**
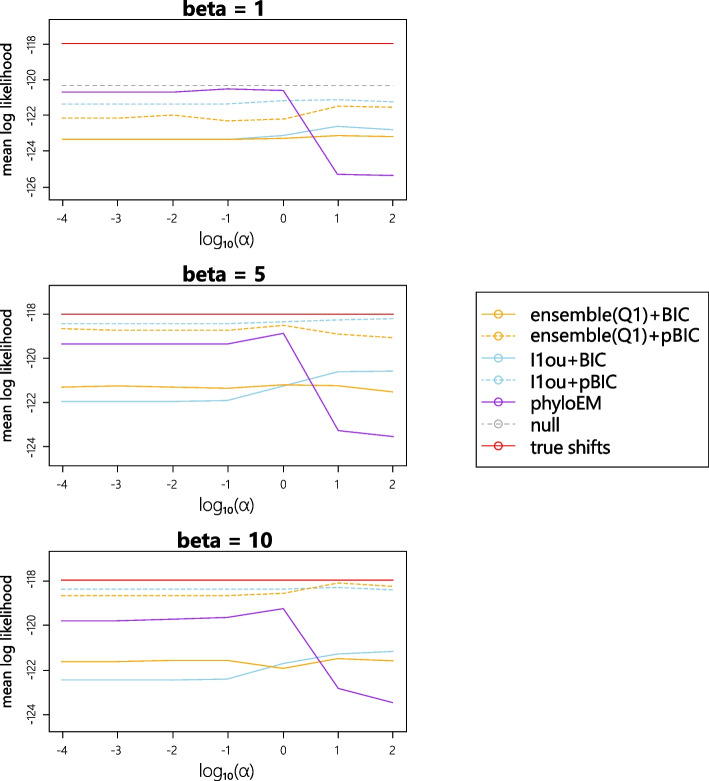
Average test log likelihood with changing estimated alpha

Figure 15 shows the prediction log likelihood with different estimated $\hat{\alpha }$. From the plots, the performance of PhylogeneticEM is influenced most by changes in the estimation of $\alpha$. Especially when the estimated $\alpha$ is too large, the method performs poorly. Ensemble methods and $\ell$1ou are more robust to misestimation of $\alpha$. Since PhlyoEM uses maximum likelihood to estimate $\alpha$, which is expected to produce more accurate estimates, robustness to misestimation is less important than for $\ell$1ou and the ensemble method, which use a very rough method to estimate $\alpha$.

### Shift in variance

**Fig. 16:**
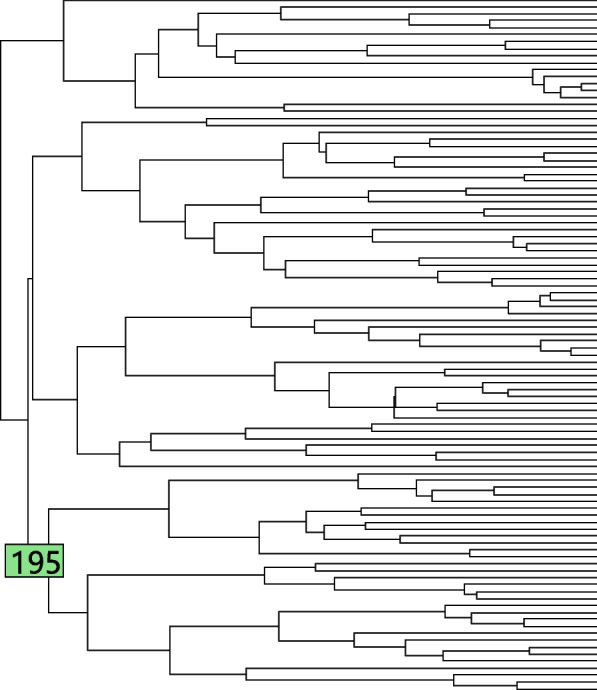
Diffusion variance parameter changes on branch 195

In this simulation study, we allow the diffusion variance parameter $\sigma ^2$ to change over the tree. In particular, we set $\sigma ^2=1$ for most of the tree, but for the part of the tree on branch 195 and below (shown in Fig. 16) we set $\sigma ^2=2, 4, 6, 8, 11$. That is, there is a shift in variance at the top of branch 195. This is a reasonable biological model, as an abrupt change of environment can bring about changes to the rate of trait evolution. We perform one simulation where there are no shifts in mean, to study the false positive rate and loss of predictive log-likelihood caused by this shift in variance.

**Fig. 17:**
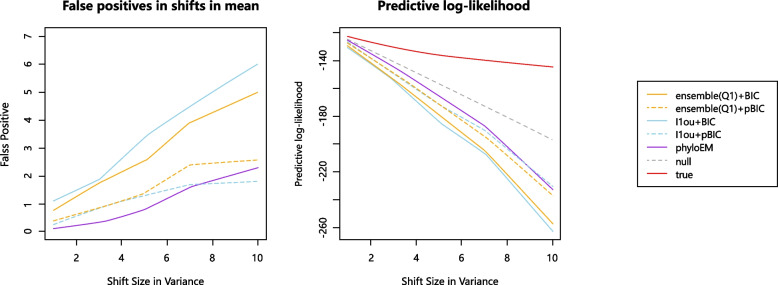
Diffusion variance parameter changes on branch 195

The left subplot of Fig. 17 shows the number of false positive shifts in optimal values detected by different methods. The right subplot of Fig. 17 shows the predictive log-likelihood of the selected models under each method. The plots show that when the diffusion variance is not constant on the tree, all of the methods will detect false positive shifts in optimal values, leading to failure to accurately predict the test data. Therefore, developing a model which incorporates shifts in variance is an interesting future research direction.

## Recommendations

We suggest applying multiple methods to each data set and comparing the results. Based on the simulation results, different methods have different strengths and we cannot say that any method outperforms the others in every situation. For example, ensemble methods and $\ell$1ou are more robust to misestimation of $\alpha$. Another example is that the methods with pBIC are the most conservative: they perform best with large signal sizes. They can detect the true shifts without introducing false positive shifts. However, they cannot give reasonable results when the signal sizes are small. The ensemble method with BIC can better capture the shifts near the leaves. PhylogeneticEM is even more conservative with small signal sizes and falls between methods with pBIC and with BIC, with large signal sizes. It is hard to tell which method and criterion is the most suitable to use in a specific task. By comparing the results of different methods, we can get the confidence level of the selected shifts. For example, the shifts which are selected by all the methods are more likely to be true. Khabbazian et al. [15] use bootstrap support to evaluate how likely the selected shifts are true. However, the bootstrap support can be influenced by biases in a particular method. By combing the results of several different methods, we can assess the confidence of particular shifts in a way that is unlikely to be influenced by the bias of any particular method.

## Conclusion

In this article, we compared the performances of several shift detection methods — $\ell$1ou, PhylogeneticEM, ensemble method — for trait evolution models. To understand the strength, weaknesses, and restrictions of different methods, we compared the performances over a large range of scenarios including both correctly specified and misspecified cases. We used three different measurements to compare the results, true positive versus false positive curve, predictive log-likelihood and Adjusted Rand Index. All three measurements give similar conclusions about the performance of the methods.

From the simulation results, when the coefficients are very small, PhylogeneticEM, $\ell$1ou+pBIC and ensemble+pBIC are very strict and tend to select nearly no shifts. In these scenarios, ensemble+BIC and $\ell$1ou+BIC perform better at detecting the small magnitude shifts. However when the coefficients are large, nearly all the methods can detect the true shifts, but $\ell$1ou+BIC and ensemble+BIC include more false positive shifts. The performances of methods are highly dependent on the criterion. A better criterion might help the methods to give very good results with varying signal sizes. Further research about appropriate model selection criteria for shift detection might be an interesting topic for future studies.

Furthermore, we compared the model performances on different shift positions in trees and different types of trees. From the results, the shifts near the leaves are the most difficult to detect and the shifts near the root are the easiest to detect. The shifts on the coalescent tree are the easiest to detect when the coefficient is small and the most difficult to detect when the coefficient is large.

We also conducted simulations in several scenarios where the model assumptions do not hold. We studied training data with measurement error; misspecified phylogenetic trees; misestimation of the parameter $\alpha$; and non-constant diffusion variance. From the simulation results, measurement error and a misspecified phylogenetic tree make shift detection more difficult and all the methods perform worse in these cases. $\ell$1ou and the ensemble method are robust to misestimation of $\alpha$. When shifts occur in the diffusion variance $\sigma ^2$, all of the methods detect the signal as many false positive shifts in optimal values, leading to failure to accurately predict the test data. Therefore, future research is needed on shift detection methods that can handle these violations of the model assumptions.

### Supplementary Information

**Additional file 1.**

## Data Availability

The tree of Anolis lizards is provided by [10]. Our R package is available at https://github.com/WenshaZ/ELPASO.
