# Supplementary material for: Evolutionary shift detection with ensemble variable selection
Source: BMC Ecol Evol. 2024 Jan 20;24:11. doi: 10.1186/s12862-024-02201-w (PMC10800078; doi:10.1186/s12862-024-02201-w)
Supplement: Supplementary file 1 — Additional file 1. [file 12862_2024_2201_MOESM1_ESM.pdf]

Appendices

A Supplemental figures

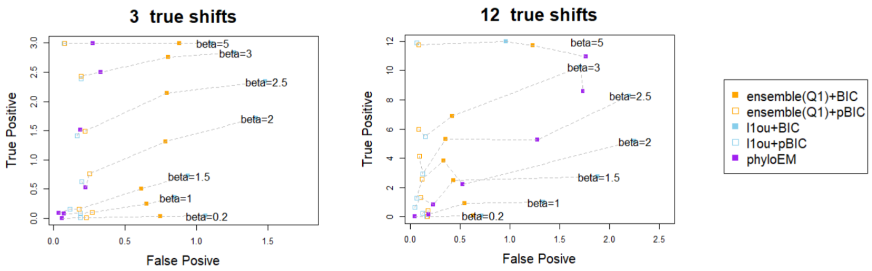

Figure 1: True positive numbers versus False positive numbers with 3, 12 shifts.

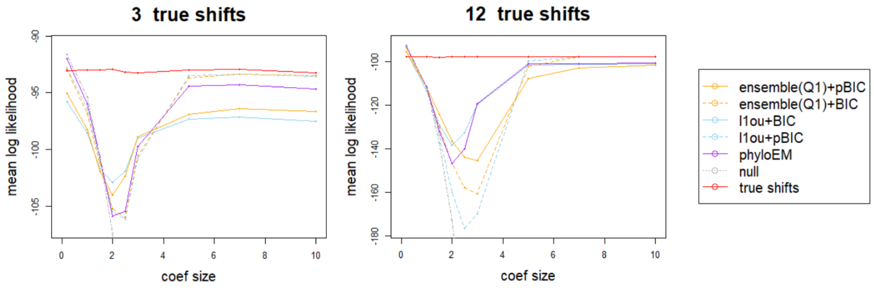

Figure 2: The mean log likelihood on 1000 test datasets (3,12 shifts)

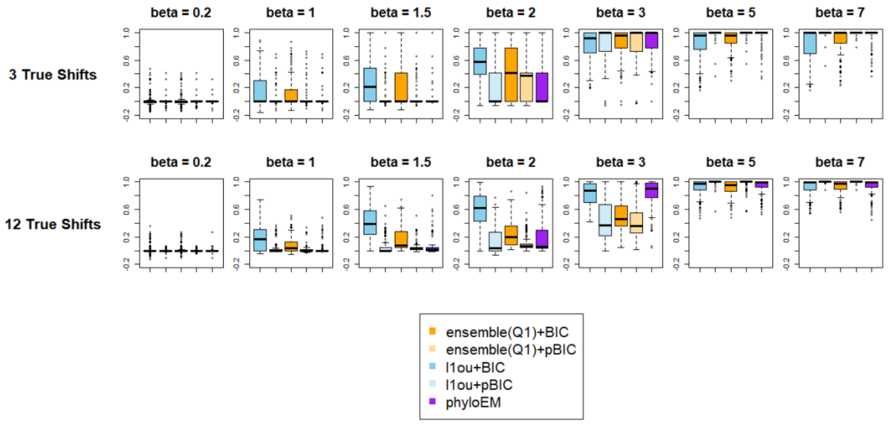

Figure 3: ARI with 3, 12 true shifts

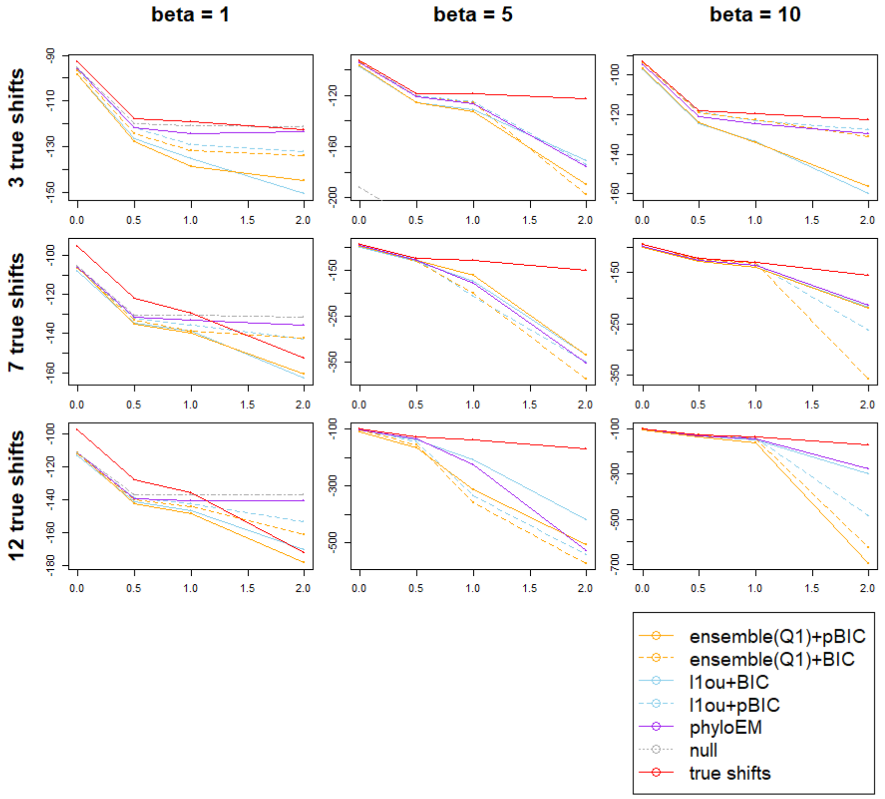

Figure 4: Average test log likelihood with parameters estimated from training data with measurement error

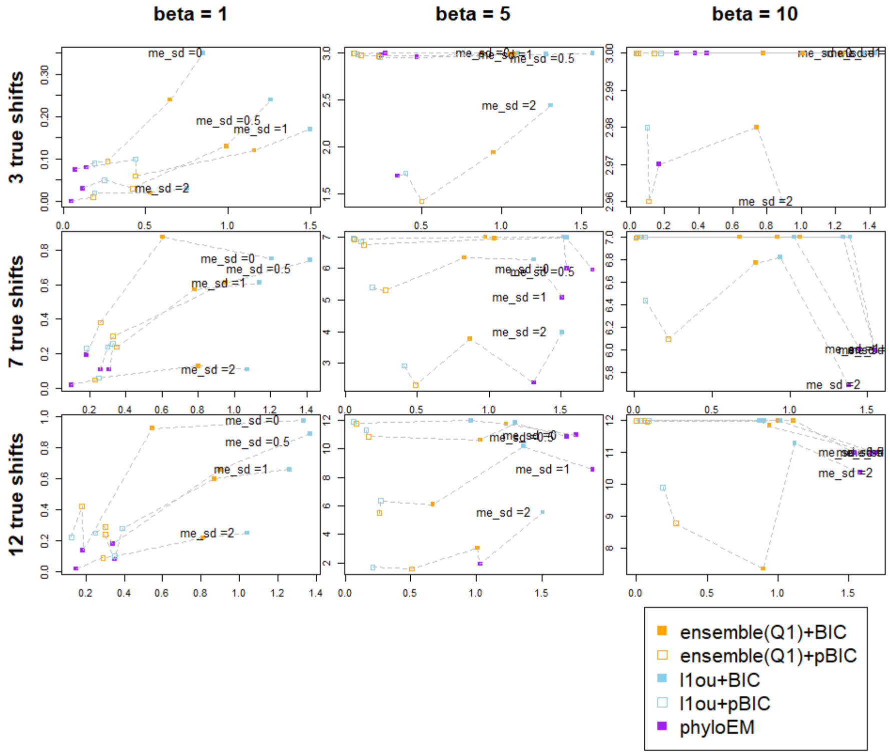

Figure 5: True positive v.s. false positive rate with applying methods on data with measurement error

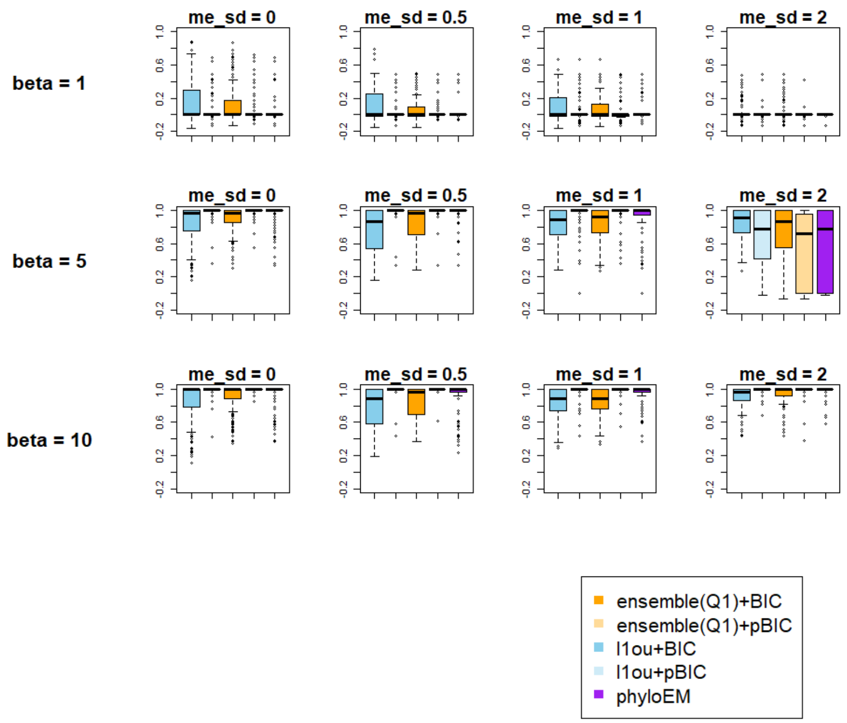

Figure 6: Adjust Rand Index plot with applying methods on data with measurement error

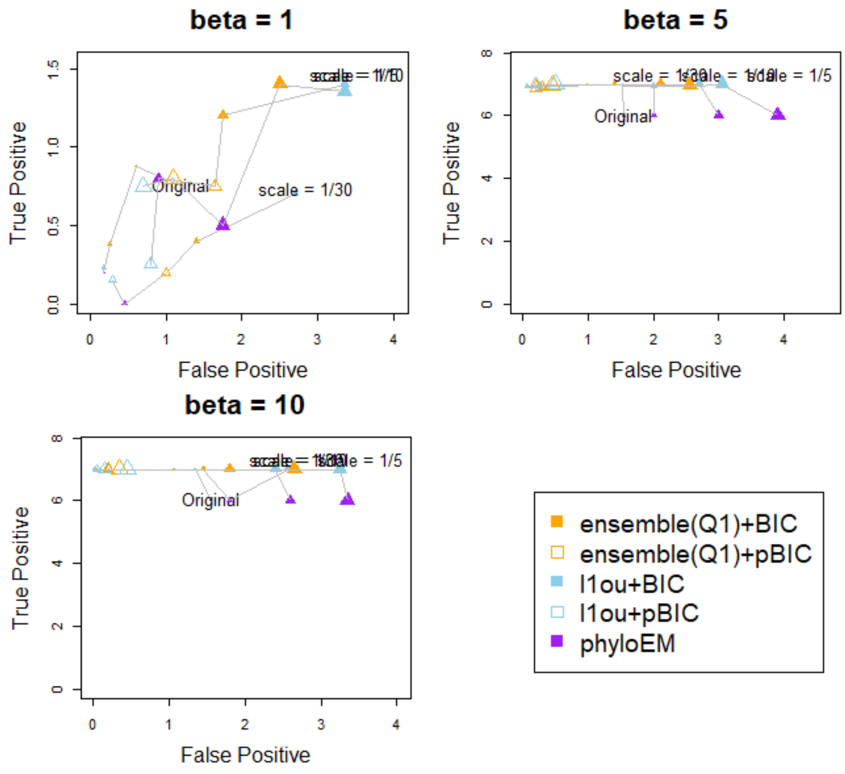

Figure 7: True positive v.s. false positive rate with applying methods on misspecified trees

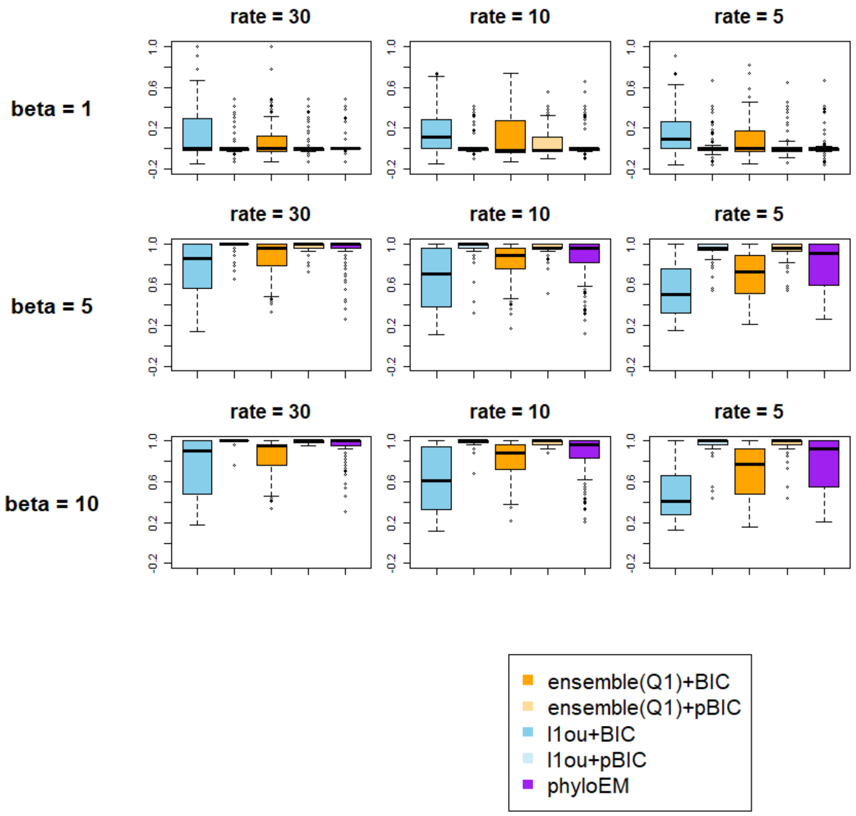

Figure 8: Adjust Rand Index plot with applying methods on misspecified trees

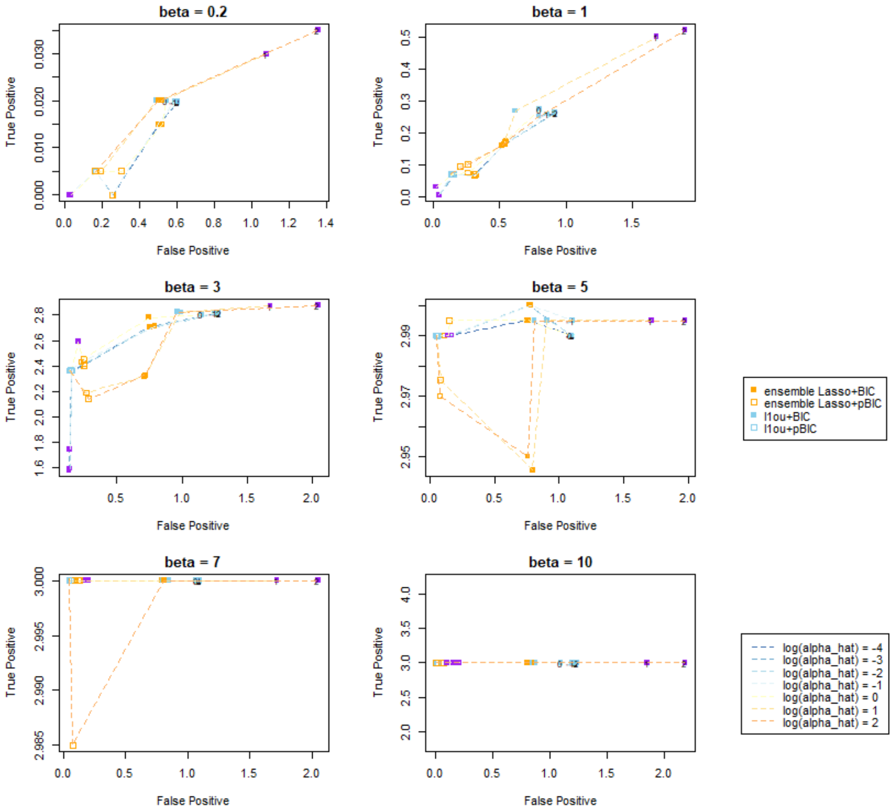

Figure 9: True positive v.s. false positive rate with changing estimated alpha

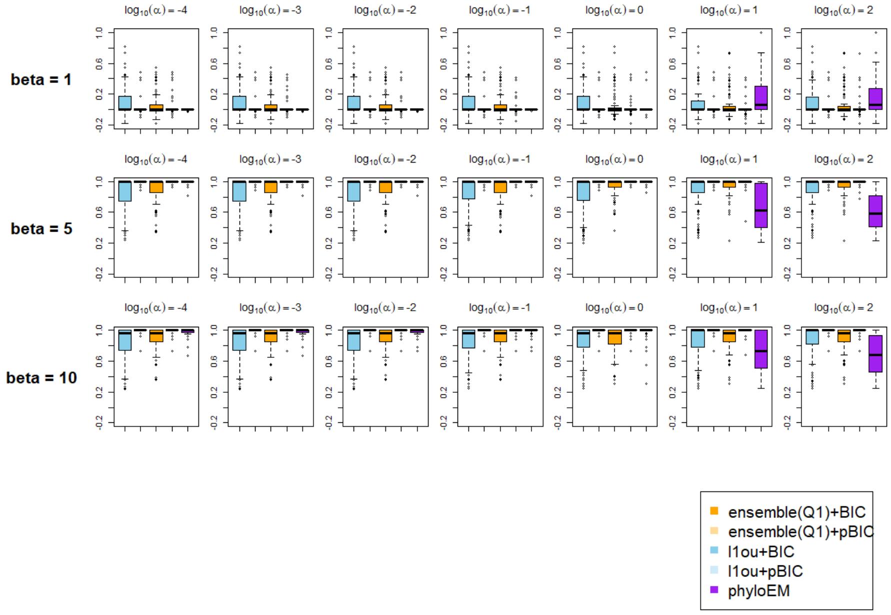

Figure 10: Adjust Rand Index plot with changing estimated alpha
